# Vibration therapy as an effective approach to improve bone healing in diabetic rats

**DOI:** 10.3389/fendo.2022.909317

**Published:** 2022-08-19

**Authors:** Maysa S. Campos, José B. Volpon, João Paulo B. Ximenez, Ana Paula Franttini, Christopher E. Dalloul, Manoel D. Sousa-Neto, Raquel A. Silva, Melissa A. Kacena, Ariane Zamarioli

**Affiliations:** ^1^ Department of Orthopaedics and Anaesthesiology, Ribeirão Preto Medical School, University of Sao Paulo, Ribeirão Preto, SP, Brazil; ^2^ Laboratory of Molecular Biology, Blood Center of Ribeirão Preto, Ribeirão Preto Medical School, Ribeirão Preto, SP, Brazil; ^3^ School of Pharmaceutical Sciences of Ribeirão Preto - University of São Paulo, Ribeirão Preto, SP, Brazil; ^4^ Department of Orthopaedic Surgery, Indiana University School of Medicine, Indianapolis, IN, United States; ^5^ School of Dentistry of Ribeirão Preto - University of São Paulo, Ribeirão Preto, SP, Brazil; ^6^ Richard L. Roudebush Veterans Affairs Medical Center, Indianapolis, IN, United States

**Keywords:** vibration, fracture healing, bony callus, diabetes mellitus, bone turnover

## Abstract

**Objective:**

To investigate the effects of vibration therapy on fracture healing in diabetic and non-diabetic rats.

**Methods:**

148 rats underwent fracture surgery and were assigned to four groups: (1) SHAM: weight-matched non-diabetic rats, (2) SHAM+VT: non-diabetic rats treated with vibration therapy (VT), (3) DM: diabetic rats, and (4) DM+VT: diabetic rats treated with VT. Thirty days after diabetes induction with streptozotocin, animals underwent bone fracture, followed by surgical stabilization. Three days after bone fracture, rats began VT. Bone healing was assessed on days 14 and 28 post-fracture by serum bone marker analysis, and femurs collected for dual-energy X-ray absorptiometry, micro-computed tomography, histology, and gene expression.

**Results:**

Our results are based on 88 animals. Diabetes led to a dramatic impairment of bone healing as demonstrated by a 17% reduction in bone mineral density and decreases in formation-related microstructural parameters compared to non-diabetic control rats (81% reduction in bone callus volume, 69% reduction in woven bone fraction, 39% reduction in trabecular thickness, and 45% in trabecular number). These changes were accompanied by a significant decrease in the expression of osteoblast-related genes (*Runx2, Col1a1, Osx*), as well as a 92% reduction in serum insulin-like growth factor I (IGF-1) levels. On the other hand, resorption-related parameters were increased in diabetic rats, including a 20% increase in the callus porosity, a 33% increase in trabecular separation, and a 318% increase in serum C terminal telopeptide of type 1 collagen levels. VT augmented osteogenic and chondrogenic cell proliferation at the fracture callus in diabetic rats; increased circulating IGF-1 by 668%, callus volume by 52%, callus bone mineral content by 90%, and callus area by 72%; and was associated with a 19% reduction in circulating receptor activator of nuclear factor kappa beta ligand (RANK-L).

**Conclusions:**

Diabetes had detrimental effects on bone healing. Vibration therapy was effective at counteracting the significant disruption in bone repair induced by diabetes, but did not improve fracture healing in non-diabetic control rats. The mechanical stimulus not only improved bone callus quality and quantity, but also partially restored the serum levels of IGF-1 and RANK-L, inducing bone formation and mineralization, thus creating conditions for adequate fracture repair in diabetic rats.

## Introduction

Diabetes is a chronic metabolic disease with a high prevalence worldwide that can lead to severe systemic complications, including cardiovascular disease, neuropathy, retinopathy, and impaired bone health (1). This relationship between diabetes and poor bone quality has been found in diabetic patients and experimental animals; type 1 diabetic patients have a significant decrease in bone mineral density, increased risk of fracture fragility, and impaired bone healing (2). Diabetes decreases bone formation due to reductions in the expression and differentiation of genes and proteins, such as osteocalcin, alkaline phosphatase, and osteoprotegerin (OPG) (3). Additionally, diabetes has also been associated with increased bone resorption by augmenting tartrate-resistant acid phosphatase (TRAP), receptor activator of nuclear factor kappa beta ligand (RANK-L), serum C terminal telopeptide of type I collagen (CTX), sclerostin, and cathepsin K levels (3, 4).

Diabetic individuals have a high risk of bone fracture due in part to imbalanced bone metabolism (5). Bone has a unique regenerative ability to heal fractures physiologically but requires a normal bone microenvironment to do so optimally. The alteration of the bone microenvironment resulting from diabetes leads to an impairment in bone healing (6, 7). Indeed, several authors have reported delayed bone healing in diabetic humans (8, 9) and animals (10–12). The primary mechanism is yet to be determined; however, several pathways have been proposed to explain the higher incidence of delayed or non-healing in diabetic individuals, including reduced blood flow and angiogenesis, an exaggerated inflammatory response, reduced collagen synthesis, and a metabolic imbalance between osteoblast and osteoclast activity (7).

Several strategies are used to treat bone healing disorders, including pharmacological avenues, autologous bone transplantation, and callus distraction. Non-invasive techniques based on mechanotransduction principles may be of great importance in improving bone cell activity. The recent description of the osteogenic effect of vibration therapy has shown it to be a promising avenue for improving bone callus formation in osteoporotic rats (13, 14). Other authors have studied the effects of vibration therapy on bone loss (13–16), but the present study is the first to investigate the effects of vibration therapy on the bone healing of experimental fractures in control and diabetic rats. We hypothesize that vibration therapy can accelerate bone healing in non-diabetic control rats and improve and even normalize the fracture union in diabetic rats.

## Methods

### Animals and experimental groups

The experimental protocol was approved by the Institutional Animal Care and Use Committee of the School of Medicine of Ribeirão Preto, University of São Paulo, Brazil.

All animals were obtained from the central animal facility of the institution. Female Wistar rats weighing approximately 200 g were housed in cages in a room with controlled humidity conditions, temperature (23 ± 1°C), and an artificial light/dark cycle of 12 hours. The experimental procedures were started after a three-day interval to allow adaptation to the laboratory environment. Animals had free access to tap water and pellet chow.

A total of 148 rats underwent fracture surgery and were assigned to four groups: (1) SHAM: weight-matched non-diabetic control rats, (2) SHAM+VT: non-diabetic control rats treated with vibration therapy, (3) DM: type 1 diabetic rats, and (4) DM+VT: diabetic rats treated with vibration therapy. Each group was subdivided into two subgroups according to the postoperative follow-up (14 and 28 days), representing two distinct phases of normal bone healing in rats, soft and hard bone callus formation, respectively (1, 4).

### Diabetes induction and bone fracture

DM and DM+VT rats received a single intravenous injection of streptozotocin (STZ) to induce diabetes. Control rats were injected with citrate buffer alone. Diabetes was diagnosed based on blood glucose concentrations (≥250 mg/dL) on two consecutive days, measured one week after STZ injection (17).

**Figure 1:**
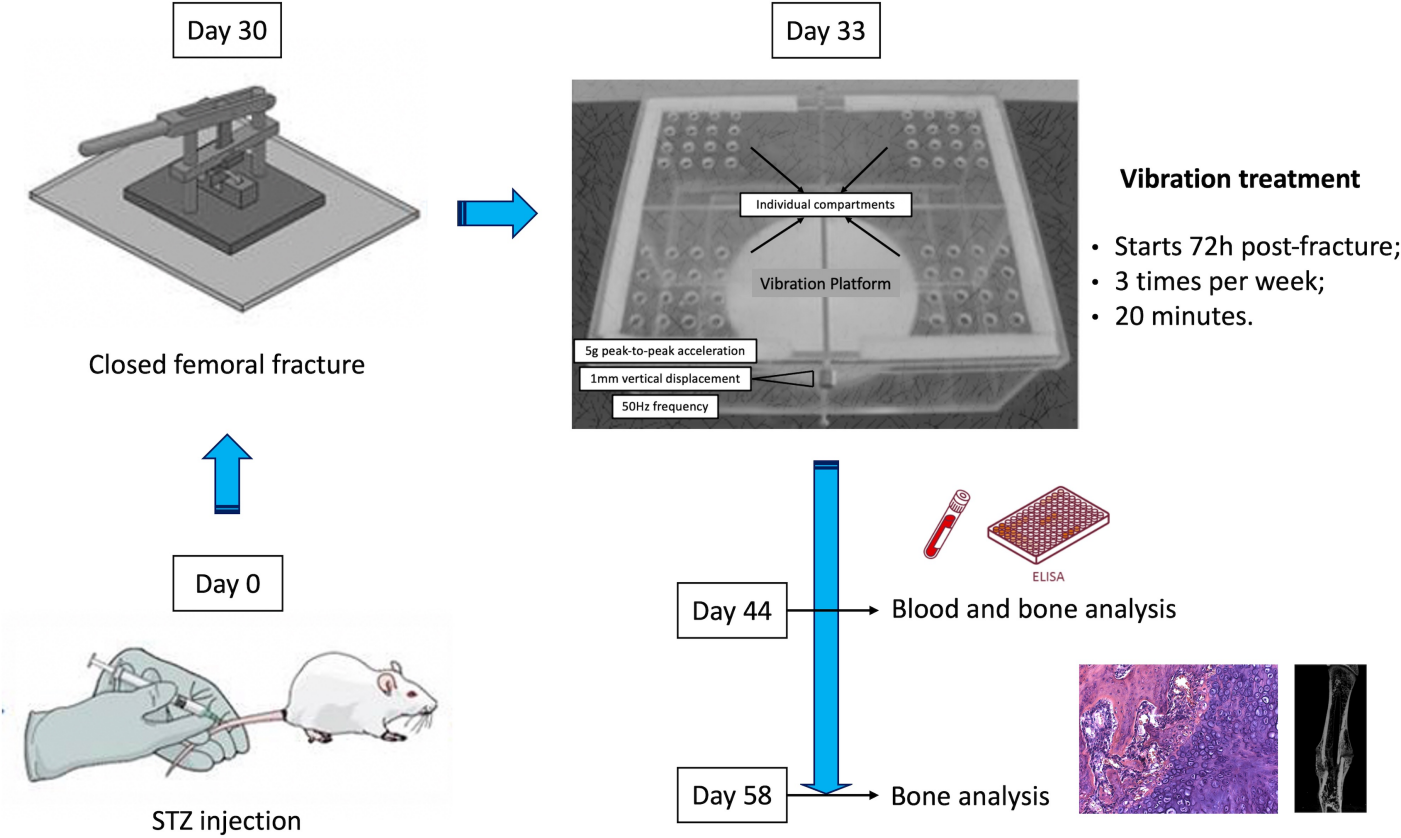
Schematic figure representing the experimental design of this study. Animals were subjected to diabetes induction by injection of streptozotocin (STZ) on study entry (day 0). On day 30, diabetic and non-diabetic rats were subjected to a mid-femur fracture. Vibration therapy (VT) was administered to SHAM+VT and DM+VT rats from day 3 post-fracture until the endpoint assessment: day 44 or day 58, in which bones and serum blood were analyzed.

Thirty days after diabetes induction or buffer injection, the animals were anesthetized, and a closed bone fracture was produced in the right mid-femur. **Figure 1** represents the experimental design of this study. For the femoral fracture procedure, rats were anesthetized with a combination (1:1) of xylazine and ketamine (0.1 mg/100 g) injected intramuscularly. The right thigh was shaved and then restrained by two metallic supports. Next, the blunted blade of a guillotine apparatus was lowered onto the mid-thigh, and a vertical force was manually applied until an abrupt decrease in resistance occurred, indicating femur fracture. Subsequently, the entire pelvic limb was cleansed with a 0.5% alcohol-based chlorhexidine antiseptic solution. An incision was then made on the lateral surface of the mid-thigh under ordinary aseptic and antiseptic surgical conditions. Next, the muscles vastus lateralis and biceps femoris were gently retracted to expose the bone fragments and the fracture site. Any fracture not located at the midshaft region or excessively fragmented was excluded. Afterward, a 1.0-mm thick Kirschner wire was introduced into the medullary canal of the proximal fragment until protruding through the trochanteric region. The fracture was then reduced under direct vision and stabilized by advancing the Kirschner wire into the medullary canal of the distal fragment to the distal condyles. The excess wire length at the trochanteric region was cut off, bent, and buried beneath the muscle. The wound tissue was closed in layers, and the skin was sprayed with a solution to avoid self-mutilation (Ecobitter, Brazil). The status of the fracture fixation was radiographically confirmed immediately after surgery and then followed-up weekly (6, 18).

Rats were allowed to recover postoperatively and were able to bear weight on their hindlimbs at immediately postoperative, exhibiting no signs of pain. Yet, they were medicated with tramadol 8 mg/kg (SC) and flunixin meglumine 2 mg/kg for five days post-surgery. The operated animals were examined daily to check for general health, wound appearance, spontaneous activity, mobility, weight-bearing, swelling, and range of motion of the knee and hip.

### Whole-body vibration therapy

Three days after bone fracture, DM+VT rats and their age-matched controls (SHAM+VT) were subjected to whole-body vibration with a peak-to-peak vertical displacement of 1 mm at a frequency of 50 Hz (5g peak-to-peak acceleration). The parameters of vibration therapy were determined based on our previous study, in which significant osteogenic effects were detected in hindlimb suspended rats (19). For this therapy, rats were placed in a vibration device consisting of a vibration desk, two alternating current engines, and a force transducer for monitoring the frequency of vibration. In addition, a plastic cage, allowing the individual location of rats, was attached to the vibration desk to maintain the animals during vibration sessions. Therapy was performed three days per week for 20 minutes for 14 and 28 days, depending on the experimental follow-up period.

On days 14 or 28 post-fracture, the rats were euthanized, blood was collected for serum bone marker analysis, and the fractured femurs were harvested in preparation for dual-energy X-ray absorptiometry (DXA) assessment, micro-computed tomography (μCT), and histological analysis.

### Serum marker assessment (enzyme-linked immunosorbent assay)

Serum samples stored at -80°C were used for the determination of insulin-like growth factor 1 (IGF-1), RANK-L, and CTX-I levels by ELISA as indicated by the manufacturer (Boster Immuno Leader and MyBioSource, USA) (SHAM: n=9; SHAM+VT: n=8; DM: n=7; DM+VT: n=7). The intra-assay variation coefficients were 5.3% for IGF-1, 5.5% for RANK-L, and <15% for CTX-I.

### DXA

Analysis was performed immediately following sacrifice using DXA with a Lunar DPX-IQ densitometer (*Lunar; software version* 4.7e, GE Healthcare, United Kingdom). The entire femur (n=7/group) was scanned, but the bone mineral density (BMD), bone mineral content (BMC), and area were only assessed over the full extent of the callus. The scanning reproducibility (4%) was assessed by the root mean square coefficient of variation.

### μCT

After DXA assessment, the femurs were scanned using a μCT device (SkyScan 1174v2; Bruker-microCT, Belgium) at 50 kV using a 0.5-mm-thick aluminum filter to optimize the contrast, a 360° rotation step of 1°, three-frame averaging, and an isotropic resolution of 26.7 μm. Images of each specimen were reconstructed (NRecon v.1.6.3) and calluses were analyzed (CTAn v.1.15.4) to determine the total callus volume (CV, in mm^3^), woven bone fraction (BV/TV, interpreted as callus mineralization, in %), and callus porosity (in %). Tissue forming callus parameters were also obtained, including trabecular thickness (in mm), number, and separation (in mm).

### Histological analysis

Seven femurs from each group were randomly selected for histological analysis.

The entire fractured femur with a small quantity of adherent soft tissue was fixed in cold 4% paraformaldehyde, decalcified in cold 10% EDTA, embedded in paraffin, sectioned at five μm, and placed on charged slides (Manco Inc., USA). Coronal sections were stained with hematoxylin and eosin (HE). Adjacent sections were stained with TRAP to identify mature osteoclasts and with Sirius red solution for collagen identification. Sections were analyzed under bright field microscopy (Axiovert; Carl Zeiss, Germany), and images were captured with a CCD camera (AxioCam MRc; Carl Zeiss, Germany) in the region of the bone callus, with magnifications of 12.5, 50, and 200x. TRAP stained slides with a magnification of 50x were also used to calculate the resorption area, in which the red color demarcated the osteoclasts, and the TRAP-positive area was measured as a percentage of the entire callus area at the fractured femur (%).

### RNA isolation and real-time PCR assessment

Total RNA was extracted from the fracture calluses (n=4/group) using SV Total RNA Isolation System (Promega) according to the manufacturer’s instruction. The expressions of genes (*Col1a1, Runx2, and Osx*) were measured using a StepOnePlus PCR machine (Applied Biosystems) and TaqMan Gene Expression Assays (Applied Biosystems). Expression levels for each gene of interest were normalized to their corresponding values of endogenous control gene GAPDH.

### Statistical analysis

The results obtained in the four groups were compared using two-way analysis of variance, followed by Tukey’s post-hoc test to detect significant interaction of diabetes and/or VT intervention. Continuous variables were expressed as the means and standard deviations (SD). P-values less than 0.05 were considered statistically significant. We also reported trending data (p<0.09) to highlight potentially interesting outcomes for future investigations. All statistical analyses were performed with RStudio 1.3 (RStudio, Inc., USA).

## Results

### Complications, mortality, and bone weight assessment

Mortality and exclusions happened throughout the study and fell into three categories: diabetes induction, maintenance, and fracture procedure (anesthesia, excessive comminution of fracture fragments, and K-wire loosening). After the streptozotocin injection, 37 rats had nondiagnostic blood glucose concentrations and were excluded from the study. Ten diabetic animals died or were euthanized due to diabetic complications, including accentuated weight loss, vision deterioration, and poor general health. Finally, twelve rats were euthanized due to the orthopaedical surgical procedure. Out of these twelve, four were euthanized during anesthesia, three were euthanized due to the excessive comminution of fracture, and five were euthanized in the postoperative period due to K-wire loosening. Therefore, our final number was 88.

**Figure 2:**
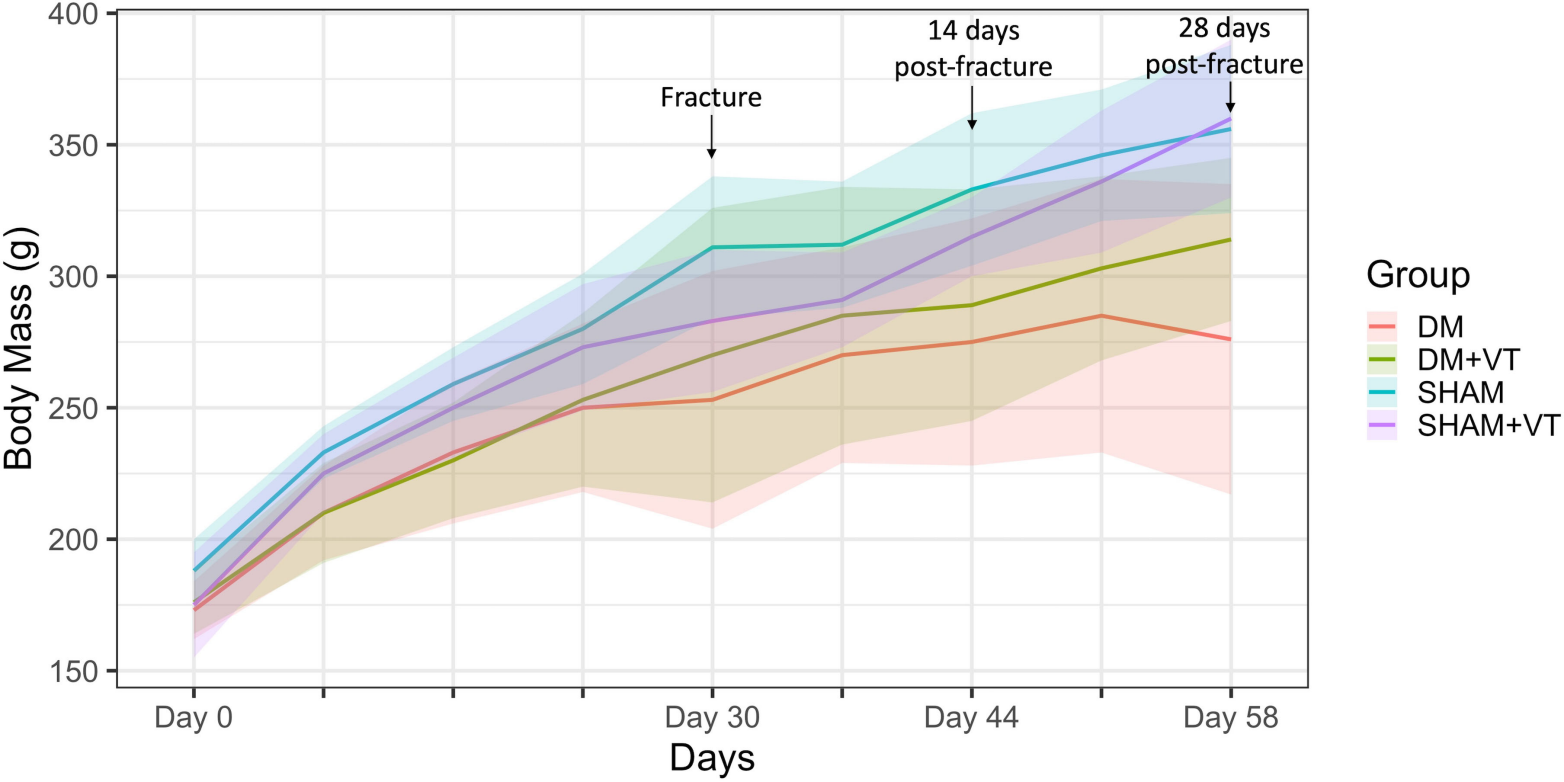
Comparison of body weight (in grams) among groups. Upon study entry (day 0, diabetes/sham induction), body weights were similar among all groups (p>0.05). On day 30, a closed femoral fracture procedure was performed. On day 14 post-fracture (day 44), the body weight was lower in diabetic rats than in sham rats (p<0.05). Similarly, on day 28 post-fracture (day 58), diabetic rats exhibited lower body weights than sham rats (p<0.05). Sample sizes are n=14/group. Lines represent the averages and shaded areas the standard deviation.

At study entry (day 0), body weights were similar among all groups (p>0.05). All rats gained weight during the observation period; however, diabetic rats gained less weight than sham rats. Thus, the final bodyweight of the diabetic rats was significantly lower than sham rats (p<0.05). Vibration therapy increased body mass gain in diabetic rats, but did not alter the body masses of sham rats; the final body masses of DM+VT rats were 7% and 16% higher than in DT rats at 14 or 28 days after fracture, respectively (p<0.05) (**Figure 2**).

### Bone quality and quantity assessment

Data will be presented as follows: (1) by detecting a significant main effect of diabetes, vibration therapy, or the interaction between them using 2-way ANOVA; (2) comparison between non-diabetic and diabetic rats (SHAM versus DM); (3) the effects of vibration therapy on diabetic rats (DM versus DM+VT); and (4) the effects of vibration therapy on non-diabetic rats (SHAM versus SHAM+VT).

### Serum marker assessment

Our 2-way ANOVA evidenced a significant main effect of diabetes on serum IGF-1 levels (p<0.00001). A significant main effect of vibration was detected for RANK-L (p=0.0003) and CTX (p=0.00002), and a significant diabetes versus vibration interaction was detected for IGF-1 (p<0.00001), with a trend observed for RANK-L (p=0.07). In the comparison between diabetic and non-diabetic rats, circulating IGF-1 was significantly reduced (-92%, p<0.05), and the CTX-I level was increased by 318% in diabetic rats in comparison with non-diabetic rats. When assessing the effects of vibration therapy on diabetic rats, we noted that the vibration treatment significantly increased the IGF-1 level by 668% (p<0.05) and decreased the RANK-L level by 19% (p<0.05) in diabetic rats compared to non-vibrated diabetic rats. When assessing the effects of vibration therapy on non-diabetic rats, we detected that the vibration treatment significantly decreased the RANK-L level by 20% and increased CTX levels by 182% in non-diabetic rats submitted to vibration when compared to the shams (p<0.05) (**Table 1**).

**Table 1:** Circulating serum bone markers assessment of IGF-1, RANK-L, and CTX-I.

	Circulating levels of IGF-1, RANK-L, and CTX-I (pg/ml) mean ± SD			
	SHAM	SHAM+VT	DM	DM+VT
IGF-1^*,‡^	3014 ± 826	1823 ± 962	246 ± 181^a^	1890 ± 652^b^
RANK-L^#,‡’^ CTX-I^#^	445 ± 127 149 ± 49	346 ± 49^c^ 420 ± 91^c^	453 ± 98 623 ± 171^a^	366 ± 80^b^ 693 ± 159

Values are expressed as mean ± S.D. IGF-1, insulin-like growth factor 1; RANK-L, receptor activator of nuclear factor kappa beta ligand; CTX-I, C terminal telopeptide of type I collagen. Sample sizes are n=9 for SHAM; n=8 for SHAM+VT; and n=7 for DM and DM+VT. Significant interactions were detected by 2-way ANOVA followed by Tukey post-hoc analyses and were designated by ^*^ when a significant main effect of diabetes was detected; by ^#^ when a significant main effect of vibration therapy was detected; or by ^‡^ when a significant diabetes x vibration therapy interaction was detected (or a trend towards significance designated by ^‡’^ when p<0.09). Significant differences were also based on: 1) diabetes (DM versus SHAM), designated by ^a^ when p<0.05 indicating a significant difference; 2) vibration therapy in diabetic rats (DM versus DM+VT), designated by ^b^ when p<0.05 indicating a significant difference; or 3) vibration therapy in non-diabetic rats (SHAM versus SHAM+VT), designated by ^c^ when p<0.05 indicating a significant difference.

### Diabetes decreased bone callus density, content, and area (DXA assessment)

**Figure 3:**
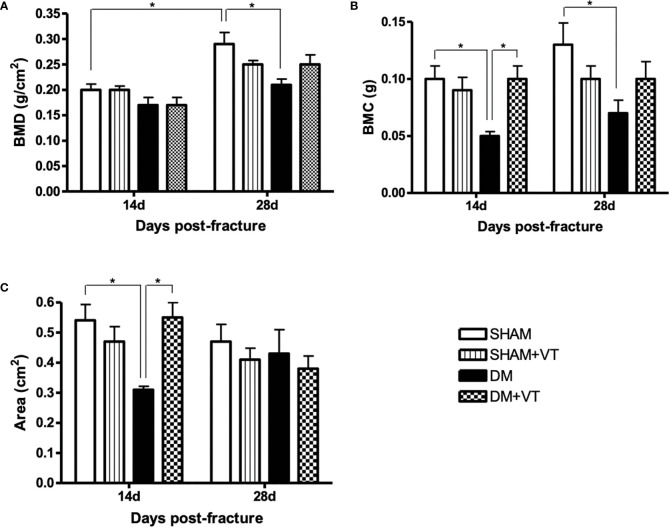
Comparison of BMD (**A**, in g/cm2), BMC (**B**, in g), and the area (**C**, in cm2) of the bone callus between each group at both 14 and 28 days post-fracture (DXA assessment). Sample sizes are n=7/group. Asterisks indicate significant differences (p < 0.05).

**Figure 3** shows a comparison of BMD (2A), BMC (2B), and area (2C) of the bone callus for each group on postoperative days 14 and 28.

At 14 days post-fracture, our 2-way ANOVA evidenced a significant main effect of diabetes in the BMD (p=0.01) and area (p=0.02), with a trend observed in the BMC (p=0.06). A significant diabetes versus vibration interaction was detected for BMC (p=0.02) and area (p=0.003). In comparing diabetic and non-diabetic rats, DM rats had significantly lower BMC and area than sham rats (p<0.05). When assessing the effects of vibration therapy on diabetic rats, we noted that the treatment significantly increased the BMC and area in DM+VT rats (p<0.05) compared to DM rats without treatment. No changes were detected between SHAM and SHAM+VT rats.

At 28 days post-fracture, our 2-way ANOVA evidenced a significant main effect for diabetes in the BMD (p=0.02) and BMC (p=0.02). Likewise, a significant diabetes versus vibration interaction was also detected in these parameters (p=0.01 for BMD and p=0.03 for BMC). Compared to non-diabetic rats, DM rats had significantly lower BMD and BMC (p<0.05). No changes were detected between vibrated and non-vibrated rats (for both diabetic and non-diabetic rats).

We further compared the two time-point DXA assessments and determined that non-diabetic rats exhibited significantly higher bone callus (with significantly higher BMD) on day 28 than on day 14 post-fracture (p<0.05).

### Bone callus microarchitecture is severely impaired by diabetes (μCT assessment)

**Figure 4:**
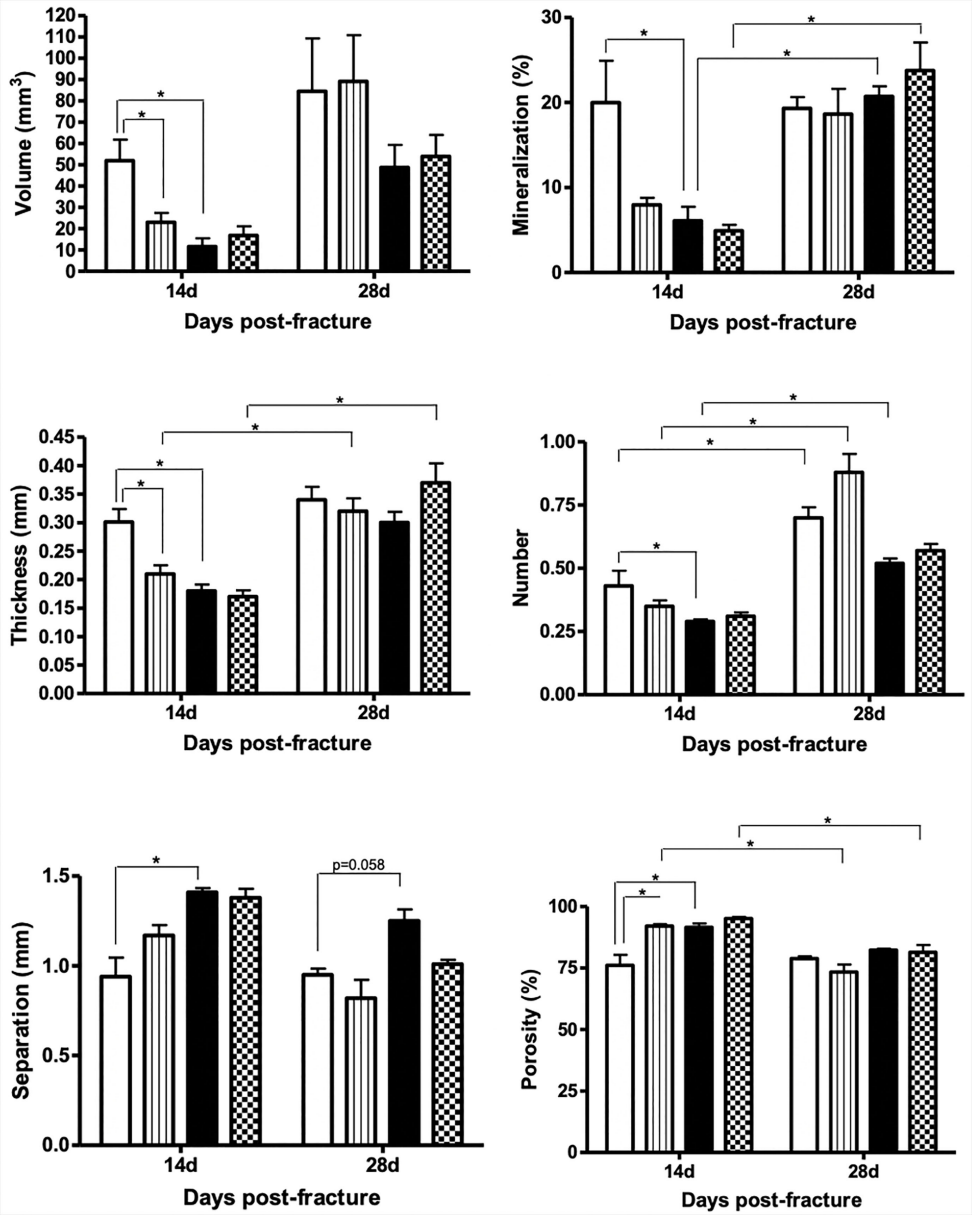
Comparison of callus volume (in mm3), mineralization (in %), thickness (in mm), number, separation (in mm), and porosity (in %) among the groups on days 14 and 28 post-fracture. Sample sizes are n = 7/group. Diabetes disrupted bone callus formation and quality by decreasing the formation-related microstructural parameters (volume, mineralization, thickness, and number) and increasing the resorption-related parameters (porosity and separation). Asterisks indicate significant differences (p < 0.05).

At 14 days after fracture, our 2-way ANOVA evidenced a significant main effect of diabetes at the callus volume (p=0.01), mineralization (p=0.02), trabecular thickness (p=0.003), trabecular separation (p=0.004), and callus porosity (p=0.02). A significant main effect of therapy was detected for callus mineralization (p=0.04), trabecular thickness (p=0.02), and callus porosity (p=0.003), with trends towards significance for callus volume (p=0.07). A significant diabetes versus vibration interaction was detected in the callus volume (p=0.01), mineralization (p=0.03), trabecular thickness (p=0.008), separation (p=0.02), and porosity (p=0.01). In the comparison between diabetic and non-diabetic rats, DM significantly impaired bone callus formation by reducing formation-related microstructural parameters (callus volume by 81%; mineralization by 69%; thickness and number of trabeculae forming callus by 39 and 45%, respectively) and by increasing resorption-related microstructural parameters (trabecular separation by 33% and callus porosity by 20%), when compared to the callus in sham rats (**Figures 3**, **4**, p<0.05). When assessing the effects of vibration therapy on diabetic rats, we noted that the vibration treatment mitigated some of the deleterious effects of diabetes and accelerated bone healing by increasing callus volume by 52% and the number of trabeculae forming callus by 35% (p>0.05) in comparison to non-vibrated diabetic rats. Although vibration therapy may exert an important osteogenic role in the bone calluses of diabetic rats, it disrupted bone healing in sham rats by decreasing callus volume by 72%, trabecular thickness by 43%, and increasing callus porosity by 25% on day 14 post-fracture (p<0.05, **Figure 4**).

On day 28 post-fracture, our 2-way ANOVA evidenced a significant main effect of diabetes on trabecular separation (p=0.02), number (p=0.003), with a trend for callus porosity (p=0.07). A trend towards significance for a main effect of therapy was detected for trabecular separation (p=0.08) and number (p=0.08). In comparing diabetic and non-diabetic rats, no significant differences were detected between any of the parameters examined by µCT; however, there was a trending increase in trabecular separation between diabetic and non-diabetic rats (35%, p=0.058). When assessing the effects of vibration therapy on diabetic rats, we noted that the beneficial effects of treatment also persisted on day 28 post-fracture by increasing trabecular thickness by 37% and decreasing trabecular separation by 21% (p>0.05). Bone calluses of vibrated sham rats did not differ from non-vibrated sham rats (p>0.05, **Figure 4**).

**Figure 5:**
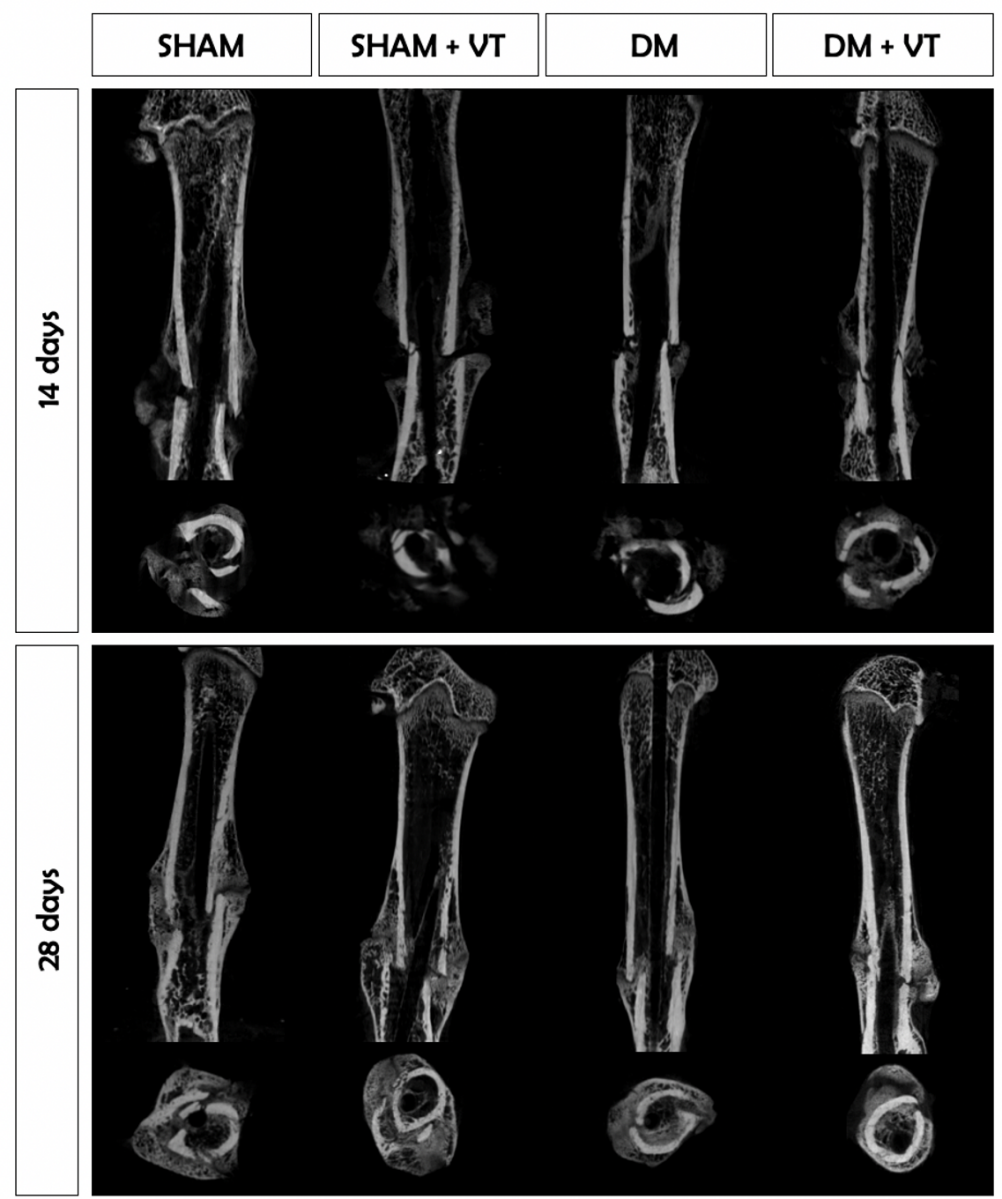
Coronal and axial tridimensional μCT images of fractured femurs. Diabetes impaired bone healing, and bone calluses were smaller and less mineralized than in sham rats 14 and 28 days post-surgery. Vibration therapy significantly increased bone callus volume and mineralization at 14 and 28 days post-surgery, but only in diabetic rats. Sample sizes are n=7/group, in which representative images from each group were selected for this figure.

We further compared the two time-point μCT assessments and detected that sham rats exhibited an increase of 56% in the number of trabeculae forming the callus on day 28 compared to day 14 post-fracture. Within the diabetic rats, calluses were 300% more mineralized, increasing by 55% in the number of trabeculae on day 28 than on day 14 post-fracture. Within the non-diabetic vibrated rats (SHAM+VT), the calluses of vibrated sham rats were 290% thicker, contained 151% more trabeculae, and were 30% less separated on day 28 than on day 14. Similarly, the calluses of vibrated diabetic rats were 450% more mineralized, 118% thicker, and 97% less separated than on day 14 post-fracture (**Figure 4**). **Figure 5** shows representative coronal and axial tridimensional μCT images of fractured femurs. Diabetes impaired bone healing, resulting in lower bone callus volumes than in sham rats. Vibration therapy significantly increased bone callus volume at both 14 and 28 days post-surgery, in diabetic rats.

### Bone callus formation is delayed by diabetes (histology)

**Figure 6:**
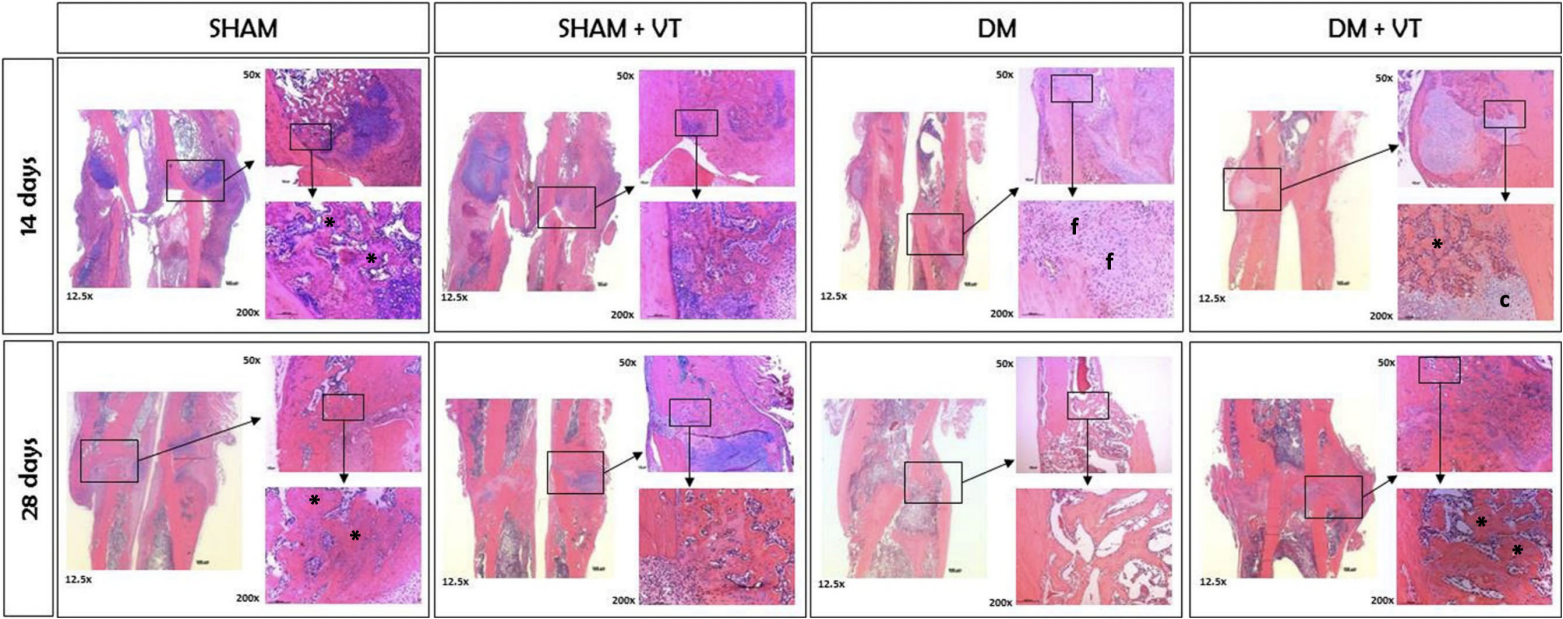
Histological slides of fractured femurs stained with HE at magnifications of 12.5, 50, and 200x. Sample sizes are n = 7/group. Diabetes induced a delay in cell proliferation and differentiation during fracture healing. At 14 days after fracture, sham rats exhibited a bone callus formed by trabeculae (represented by *), while diabetic rats only showed connective tissue with fibroblasts (represented by f). The delay persisted in the later stage of healing, where sham rats had dense trabeculae forming a bone callus (*), and diabetic rats only showed thin and well-spaced trabeculae. Vibration therapy accelerated cell differentiation during fracture healing in diabetic rats in both stages but did not cause changes in normal bone healing. Calluses were formed by cartilaginous tissue (c) and newly formed trabeculae (*) on day 14 and thick and dense trabeculae (*) on day 28.

**Figure 7:**
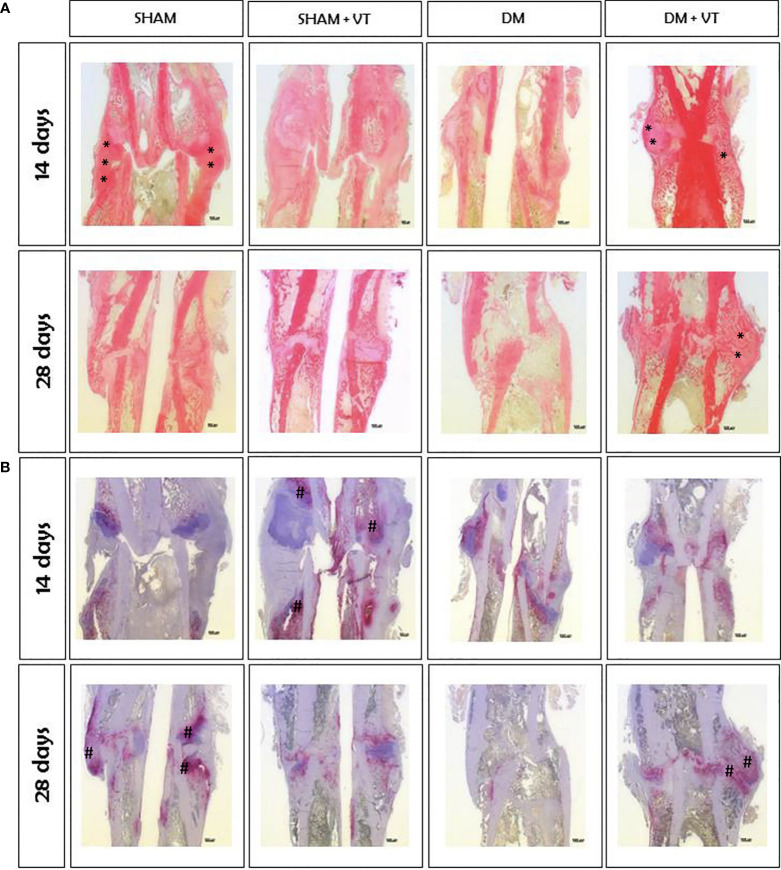
Histological slides of fractured femurs stained with Sirius red solution **(A)** and TRAP **(B)**, with a magnification of 12.5x. **(A)**: Collagen deposition (shown by asterisk marks) in the bone calluses of diabetic rats was significantly lower than in sham rats. Vibration therapy increased collagen deposition in bone calluses of diabetic rats but not in sham rats. **(B)**: Osteoclastic activity (shown by hashtag marks) (#) in diabetic rats was higher than in sham animals on day 14 after fracture but lower on day 28. Vibration therapy increased osteoclastic activity in bone calluses of diabetic rats on day 28 post-fracture but did not exert a desirable effect in control rats. Sample sizes are n=7/group, in which representative images from each group were selected for this figure. Asterisks (*) indicate collagen deposition.

At 14 days after bone fracture, sham rats showed bone calluses formed by cartilaginous tissue and newly formed trabeculae (represented by asterisks in **Figure 6**), abundant collagen deposition (represented by asterisks in **Figure 7A**), and low osteoclastic activity (**Figure 7B**). Diabetic rats, however, exhibited only connective tissue with fibroblasts (represented by “f” in **Figure 6**), little collagen deposition (**Figure 7A**), and intense osteoclastic activity (represented by hashtags in **Figure 7B**). Measuring TRAP-positive area resulted in a significant 64% increase in the resorption activity on day 14 post-fracture at the calluses in the diabetic rats compared to the shams (p=0.002, **Table 2**). This delay in the healing process persisted 28 days after bone fracture; sham rats displayed a bone callus composed of very organized, thick, and dense trabeculae (asterisks, **Figure 6**) associated with high osteoclastic activity and represent the remodeling phase of bone healing (hashtags, **Figure 7B**). Contrastingly, at this later time point, diabetic rats only showed thin, unorganized, and dispersed trabeculae (**Figure 6**), with little osteoclastic activity (**Figure 7B**). Notably, our TRAP stained sections revealed a significant 40% decrease in the resorption activity on day 28 post-fracture at the calluses in the diabetic rats compared to the shams (p=0.004, **Table 2**).

**Table 2:** Assessment of resorption area by TRAP-positive area calculation at the fractured calluses.

	TRAP-positive area at the fracture callus (%) mean ± SD			
	SHAM	SHAM+VT	DM	DM+VT
Day 14	1.82 ± 0.51	2.97 ± 0.92^c^	2.99 ± 0.49^a^	2.20 ± 0.63^b^
Day 28^’^	2.90 ± 0.50	1.98 ± 0.64^c^	1.73 ± 0.58^a^	2.86 ± 0.77^b^

Values are expressed as mean ± S.D. TRAP, tartrate-resistant acid phosphatase. Sample sizes are n=7/group. Significant differences were also based on: 1) diabetes (DM versus SHAM), designated by ^a^ when p<0.05 indicating a significant difference; 2) vibration therapy in diabetic rats (DM versus DM+VT), designated by ^b^ when p<0.05 indicating a significant difference; or 3) vibration therapy in non-diabetic rats (SHAM versus SHAM+VT), designated by ^c^ when p<0.05 indicating a significant difference.

Conversely, vibration therapy when administered to the shams resulted in increased bone resorption compared to the non-treated rats. On day 28, TRAP-positive area was significantly lower in the diabetic group than in the Shams, which was restored by vibration therapy treatment (DM+VT versus DM). Conversely, vibration therapy when administered to the shams resulted in decreased bone resorption compared to the non-treated rats. On day 14 post-fracture, TRAP-positive area was significantly higher in the diabetic group than in the Shams, which was restored by vibration therapy treatment (DM+VT versus DM).

Vibration therapy accelerated bone healing in diabetic rats. Within 14 days of bone fracture, vibrated diabetic rats exhibited a bone callus with cartilaginous tissue (represented by “c” in **Figure 6**), newly formed trabeculae, and collagen deposition (represented by asterisks in **Figures 6**, **7A**) instead of only connective tissue seen in diabetic rats with no vibration therapy. Furthermore, vibration therapy on day 14 post-fracture significantly decreased the resorption area by 26% compared to the diabetic rats without treatment (p=0.04, **Table 2**) with values comparable to those noted in the sham rats (p=0.28). This osteogenic effect persisted 28 days after fracture. Thicker, more organized, and denser trabeculae, higher collagen deposition, and increased osteoclastic activity were seen in vibrated diabetic rats compared to non-vibrated diabetic rats (**Figures 6**, **7**). Of note, vibration therapy on day 28 post-fracture significantly increased the resorption area by 65% compared to that observed in the diabetic rats without treatment (p=0.02, **Table 2**) with values comparable to those seen in the sham rats (p=0.93). The osteogenic effects of vibration therapy were not observed in sham vibrated rats, although vibration therapy increased osteoclastic activity 14 days after bone fracture and reduced it on day 28 (**Figure 7B**), both undesirable features. Our TRAP stained sections further revealed a significant 63% increase in TRAP-positive area in the sham-vibrated rats compared to those with no vibration on day 14 post-fracture (p=0.02, **Table 2**), and a 32% reduction on day 28 (p=0.02, **Table 2**).

### Osteoblast differentiation is impaired by diabetes

Unfortunately, the samples for the SHAM+VT group on day 14 post-fracture were excluded from the PCR analysis due to an unexpected storage incident with the freezer, which could highly impair gene quality.

**Figure 8:**
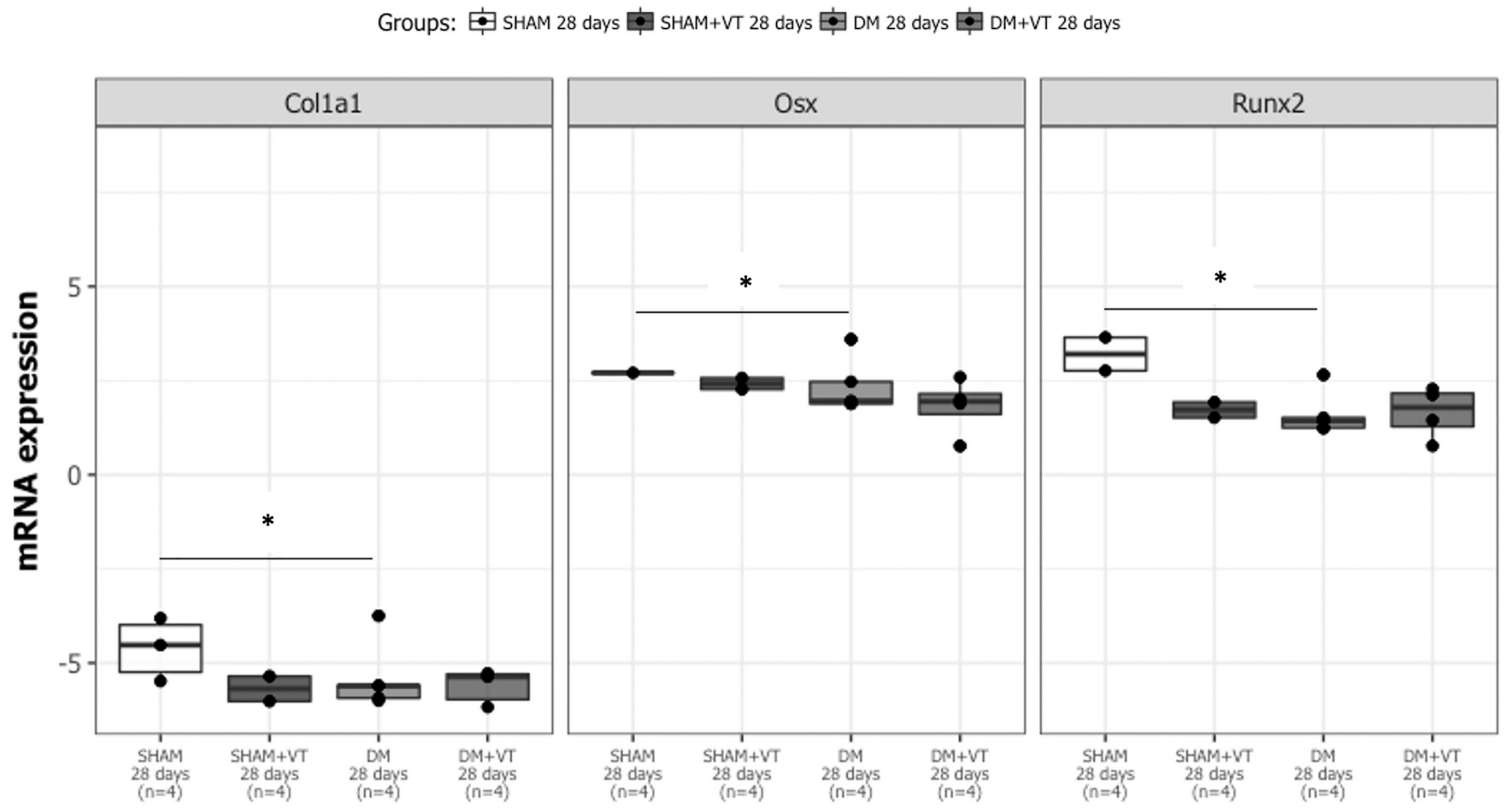
Diabetes leads to down expression of osteoblast-related genes (Col1a1, Runx2, and Osx) on day 28 post-fracture. Sample sizes are n = 4/group. Asterisks (*) indicate significant difference (p<0.05).

On day 28 post-fracture, our 2-way ANOVA evidenced a significant main effect of diabetes on *Col1a1* (p=0.0002)*, Runx2* (p=0.0008), and *Osx* (p=0.01) expression when comparing diabetic and non-diabetic rats. In fact, diabetic rats exhibited lower expression of osteoblast-related genes than non-diabetic animals (p=0.00004 for *Col1a1*; p=0.008 for *Runx2*, and p=0.02 for *Osx* **Figure 8**). Also, a trend towards significance for the *Osx* expression was detected in comparing non-diabetic sham rats and non-diabetic sham rats submitted to vibration (SHAM vs. SHAM+VT, p=0.08).

## Discussion

Diabetes and osteoporosis are two related conditions of great interest, and their incidence is currently increasing worldwide. In 2015, 422 million people across the world were diagnosed with diabetes. Additionally, 1.5 million diabetic individuals die every year from diabetic complications (20).

Several human studies (8, 9) and experimental investigations (6, 7, 10–12, 17, 21, 22) have documented the association between diabetes and disruption in bone healing. In normal secondary bone healing, four distinct phases are required to heal bone tissue: inflammation, soft callus formation, hard callus formation, and remodeling (4, 23, 24) (25). A hematoma forms between bone fragments during the inflammatory stage, which releases multiple growth factors, including platelet-derived growth factor (PDGF) and transforming growth factor b (TGF-b), into the fracture site (10, 24).

Although the exact mechanism leading to delayed bone healing or non-union in diabetic humans and animals is yet to be determined, diabetes is associated with reduced cellular proliferation, collagen deposition, and mechanical properties (1, 6, 7, 26). Previous studies in diabetic rats have demonstrated inadequate biological signals in an early phase of bone healing (2, 4, and 7 days), with decreased levels of PDGF, TGF-b, IGF-1, and vascular endothelial growth factor (VEGF) at the fracture site (10). Such disturbances in the growth factor signaling cascade result in the impaired proliferation and differentiation of mesenchymal cells into osteoblasts and decreased angiogenesis (7). This study observed that diabetic rats showed decreased IGF-1 levels associated with increased circulating CTX-I levels, which may have resulted in decreased collagen deposition and a delay in the differentiation of bone cells to form a bone callus 14 days after fracture. By this stage in the normal healing process, the bone callus was expected to be formed by cartilaginous tissue and immature trabecular bone; however, the bone callus of diabetic rats was mainly formed by connective tissue. A similar delay in cell differentiation was also observed 28 days after bone fracture. Specifically, in normal healing at 28 days after fracture, the bone callus should be mainly formed by thick and dense trabeculae. Instead, diabetic rats showed a bone callus composed of thin and disperse trabeculae, which may be related to the lower gene expression of *Col1a1, Runx2*, and *Osx*, confirming a substantial impairment in bone formation. Consequently, the bone callus of diabetic rats was less dense and likely weaker. These results highlight the need for non-invasive therapeutic modalities with the ability to reverse compromised fracture healing to be used as adjuvant therapies to treat diabetic patients.

Previous studies have examined the effects of vibration therapy on improving bone healing in rats with several osteometabolic disturbances (14, 27–33). Although no studies have investigated the effects of such therapy on bone healing in diabetic rats, Weinheimer et al. determined an important angiogenic effect of vibration therapy on wound healing in diabetic mice (34). Thus, we hypothesized that such mechanical stimuli associated with sensory-motor disturbances due to wave vibration might accelerate bone healing in control rats and improve and perhaps even normalize the consolidation observed in diabetic rats.

Although several studies have investigated the effects of vibration therapy on bone healing, the protocols are exceedingly divergent. Indeed, some authors have used low-magnitude vibration protocols, with peak acceleration lower than or equal 0.3 g (14, 28–30, 32, 35), others have adopted protocols with 3 g (27, 36), 4 g (33), 7.9 g (37), and even as high as 16 g (31). Additionally, results may also differ within the same study, using the same gravitational force, but different frequencies (38). A systematic review of 19 articles investigating whole-body vibration and fracture healing found no serious complications or side effects of this treatment, but further investigation and safety standards are needed before whole-body vibration can be translated to fracture patients (39). Moreover, we previously reported a significant osteogenic effect using a 5 g whole-body vibration protocol in rats with bone loss due to disuse (19). Therefore, the same vibration protocol was used in the current study, in which we found that cell proliferation at the bone callus was significantly higher in diabetic rats treated with vibration therapy than in non-treated diabetic rats.

Vibration therapy increased the level of circulating IGF-1, which was also associated with a reduction in RANK-L. These findings may explain the increased cell proliferation in chondrogenesis, trabecular formation, and collagen deposition observed in the bone callus of diabetic rats, which are associated with increased mineralization and volume. Though a detailed description of the cellular and molecular pathways involved remains to be elucidated, whole-body vibration therapy appears to promote micromovements at the fracture site. These micromovements result in a bone cell response and may increase and accelerate differentiation and expression toward bone repair (28). Furthermore, a recent study evidenced the important role of skeletal muscle in the healing stimulation due to vibration therapy, whose efficacy was reduced by the presence of sarcopenia in mice (40). Whole-body vibration may increase the expression of genes related to osteoblastogenesis and upregulate growth factors related to bone formation (i.e., IGF-1), thus accelerating bone healing in diabetic rats. Furthermore, a recent animal study evidenced that vibration therapy can restore the impaired inflammatory response and enhance callus formation in OVX-induced osteoporotic fracture healing by activating the p38 mitogen-activated protein kinases (p38 MAPK pathway). The p38 MAPK pathway is important for normal immune and inflammatory response, as well as it is essential for skeletogenesis and osteoblast differentiation, and may triggered the osteogenic effects of vibration therapy in the diabetic rats of this study (41).

Unexpectedly, the vibration regimen was not found to accelerate bone healing in sham rats and impaired critical callus microstructural parameters at the 14-day time point. Similar findings were recently reported in healthy mice submitted to low-intensity vibration therapy, in which the ratio of bone-to-tissue volume and the tissue volume density were lower for the vibrated group than control on week three post-fracture (42). Likewise, a recent study concluded that local application of vibration did not offer additional benefit to the healing of rabbit calvarial defects treated with grafting materials and membranes (36).

We posit that a substantial deficit in the fracture healing process is required for vibration therapy to produce a noticeable benefit.

The diabetic rats in this study were deprived of treatment such as insulin replacement as we aimed to assess the effects of type 1 diabetes on bone healing. Although such a condition may not be clinically relevant as patients will be treated once they have a confirmed diagnosis, further understanding of fracture healing in untreated diabetic rats is still of great interest in diabetes research. This understanding will allow us to assess the effects of insulin replacement and other treatments on impaired bone healing due to diabetes in later studies. It should be mentioned that the rats used in this study were seven weeks old, representing young adults, which is the most prevalent age among individuals with type 1 diabetes (43). Likewise, the high attrition rate observed in this study should also be highlighted and discussed. We have carefully followed the Guidelines for Animal Welfare and strictly followed the criteria for humane endpoint. We believe the high attrition rate may only be reduced by controlling the hyperglycemia of the diabetic rats. However, in this study we aimed to assess the effects of uncontrolled diabetes. A further alternative to lower the attrition rate could rely on reducing the postoperative endpoint assessments.

To the best of our knowledge, this study is the first to investigate the effects of vibration therapy on bone healing in diabetic rats. Our results indicate that vibration therapy significantly counteracted several of the deleterious effects of diabetes on bone healing, suggesting the therapy’s potential as an important osteogenic approach to stimulate bone healing without adding external substances into the bone cell environment.

## Data availability statement

The raw data supporting the conclusions of this article will be made available by the authors, without undue reservation.

## Ethics statement

The animal study was reviewed and approved by Animal Care and Use Committee of the School of Medicine of Ribeirão Preto, University of São Paulo.

## Author contributions

MC, JV, and AZ designed the experiments. MC and AF performed the experiments. MC, JV, CD, MS-N, RS, JX, MK, and AZ analyzed the data and revised the manuscript. AZ composed the research proposal, conceived and designed the experiments, discussed all of the results, and wrote the manuscript. All authors contributed to the article and approved the submitted version.

## Funding

This study was funded by the São Paulo Research Foundation (FAPESP) and Coordination for the Improvement of Higher Education Personnel (CAPES). Results of this work were supported by NIH R01 AG060621 (MAK) and with resources and the use of facilities at the Richard L. Roudebush VA Medical Center, Indianapolis, IN: VA Merit #BX003751 (MAK).

## Conflict of interest

The authors declare that the research was conducted in the absence of any commercial or financial relationships that could be construed as a potential conflict of interest.

## Publisher’s note

All claims expressed in this article are solely those of the authors and do not necessarily represent those of their affiliated organizations, or those of the publisher, the editors and the reviewers. Any product that may be evaluated in this article, or claim that may be made by its manufacturer, is not guaranteed or endorsed by the publisher.
